# Supplementary material for: Randomized Controlled Trials to Treat Obesity in Military Populations: A Systematic Review and Meta-Analysis
Source: Nutrients. 2023 Nov 14;15(22):4778. doi: 10.3390/nu15224778 (PMC10674729; doi:10.3390/nu15224778)
Supplement: Supplementary file 1 [file nutrients-15-04778-s001.zip › nutrients-2642176-supplementary.pdf]

Supplementary Materials

Text S1. Full search strategy

PubMed:

("obes*" OR "adiposity" OR "overweight") AND ("militar*" OR "military personnel" OR "army" OR "navy" OR "military force*" OR "air force*" OR "soldier*") AND ("treatment*" OR "clinical trial*" OR "psychotherapy" OR "psychological therapy" OR "CBT" OR "cognitive behavioral therapy" OR "pharmacological" OR "medication*" OR "drug*")

Web of Science:

("obes*" OR "adiposity" OR "overweight") AND ("militar*" OR "military personnel" OR "army" OR "navy" OR "military force*" OR "air force*" OR "soldier*") AND ("treatment*" OR "clinical trial*" OR "psychotherapy" OR "psychological therapy" OR "CBT" OR "cognitive behavioral therapy" OR "pharmacological" OR "medication*" OR "drug*")

PsychINFO:

(obes* OR adipos* OR overweight*) AND (militar* OR military person* OR arm* OR nav* OR military-force* OR air-force* OR soldier*) AND (treatment* OR clinical trial* OR psychotherap* OR psychological therap* OR CBT OR cognitive behavioral therap* OR pharmacological* OR medication* OR drug*)

Text S2. Narrative synthesis of studies not included in meta-analyses

A total of three studies investigated BW or BMI outcome of weight loss intervention in military population, but due to the values being unobtainable they were not included in the meta-analysis [1-3]. All these studies focused on behavioural and lifestyle modification and reported mixed findings. 12-months behavioural interventions specifically designed for veterans with mental illness and antipsychotic medication-associated obesity consisting of classes and individual nutritional counselling with a dietitian showed a significant decrease in BMI for the treatment group compared to controls [1]. Despite this, 12-weeks study on overweight US active-duty military personnel focusing on lifestyle change using a pedometer pointed out a significant decrease in BMI from baseline to post-test, even if this change weren't significantly different between treatment and control groups [2]. Finally, a study examined the effectiveness of a second-year weight loss intervention for obese veterans based on lifestyle modification compared to usual care program highlighting how although the participants of the treatment group initially had greater weight loss, treatment was not sufficient to sustain weight loss through the second year [3].

In addition, two studies designed randomized-controlled trials to treat obesity in military personnel but were not include in the meta-analyses due to data not available because of still in progress studies [4,5]. One study describes an 8-weekly cohort-randomized controlled trial of acceptance and commitment therapy (ACT) to enhance a Navy's weight management program and aims to determine the effectiveness of the intervention in

the treatment group compared to controls. The primary outcome will be the body weight, as well as the BMI, the body fat percentage, problematic eating, and quality of life will be measured as secondary outcomes [4]. The second studies address to determine the effectiveness of a randomized control trial evaluating a Cognitive Behavioral Therapy based on psychoeducation, coping skills, and experiential learning for veterans with binge eating disorder and obesity. Primary outcomes will be the evaluation of reduction of binge eating, body weight measurement, and energy intake over 5-months of treatment and after 6-month follow-up [5].

Table S1. Scottish Intercollegiate Guidelines Network (SIGN) Methodology Checklist for Randomized Controlled Trials [6].

SIGN	Methodology Checklist 2: Controlled Trials	
Study identification (*Include author, title, year of publication, journal title, pages*)		
Guideline topic:	Key Question No:	Reviewer:

Before completing this checklist, consider:

1. Is the paper a **randomised controlled trial** or a **controlled clinical trial**? If in doubt, check the study design algorithm available from SIGN and make sure you have the correct checklist. If it is a **controlled clinical trial** questions 1.2, 1.3, and 1.4 are not relevant, and the study cannot be rated higher than 1+
2. Is the paper relevant to key question? Analyse using PICO (Patient or Population Intervention Comparison Outcome). IF NO REJECT (give reason below). IF YES complete the checklist.

Reason for rejection: 1. Paper not relevant to key question ☐ 2. Other reason ☐ (please specify):

**SECTION 1: INTERNAL VALIDITY**		
In a well conducted RCT study…		***Does this study do it?***
1.1	The study addresses an appropriate and clearly focused question.	Yes ☐ No ☐ Can't say ☐
1.2	The assignment of subjects to treatment groups is randomised.	Yes ☐ No ☐ Can't say ☐
1.3	An adequate concealment method is used.	Yes ☐ No ☐ Can't say ☐
1.4	The design keeps subjects and investigators 'blind' about treatment allocation.	Yes ☐ No ☐ Can't say ☐
1.5	The treatment and control groups are similar at the start of the trial.	Yes ☐ No ☐ Can't say ☐
1.6	The only difference between groups is the treatment under investigation.	Yes ☐ No ☐ Can't say ☐
1.7	All relevant outcomes are measured in a standard, valid and reliable way.	Yes ☐ No ☐ Can't say ☐

1.8	What percentage of the individuals or clusters recruited into each treatment arm of the study dropped out before the study was completed?	Treatment Group: %<br>Control Group: %
1.9	All the subjects are analysed in the groups to which they were randomly allocated (often referred to as intention to treat analysis).	Yes ☐ No ☐<br>Can't say ☐ Does not apply ☐
1.10	Where the study is carried out at more than one site, results are comparable for all sites.	Yes ☐ No ☐<br>Can't say ☐ Does not apply ☐

**SECTION 2: OVERALL ASSESSMENT OF THE STUDY**		
2.1	How well was the study done to minimise bias?<br>*Code as follows:*	High quality (++)☐<br>Acceptable (+)☐<br>Low quality (-)☐<br>Unacceptable – reject 0 ☐
2.2	Taking into account clinical considerations, your evaluation of the methodology used, and the statistical power of the study, are you certain that the overall effect is due to the study intervention?	
2.3	Are the results of this study directly applicable to the patient group targeted by this guideline?	
2.4	**Notes.** Summarise the authors' conclusions. Add any comments on your own assessment of the study, and the extent to which it answers your question and mention any areas of uncertainty raised above.	

Table S2. Quality assessment of the studies included in the meta-analysis and systematic review using the SIGN Methodology Checklist for Randomized Controlled Trials

	1. Internal Validity										2. Overall Assessment			3. Rating
**Study**	**1.1**	**1.2**	**1.3**	**1.4**	**1.5**	**1.6**	**1.7**	**1.8**	**1.9**	**1.10**	**2.1**	**2.2**	**2.3**	**(++), (+), (-), (0)**
Damschroder et al., [7]	*	*				*	*	24%<br>25%	*	*	++	*	*	++
Dennis et al.,[8]	*	*			*	*	*	0%<br>0%	*	-	+	*	*	+
Erickson et al., [1]	*	*			*	*	*	14%<br>15%	*	*	+	*	*	++
Evans-Hudnall et al.,[9]	*	*				*	*	0%<br>0%	*	-	+	*	*	+
Goldberg et al., [10]	*	*			*	*	*	43%<br>27%	*	*	++	*	*	++
Hoerster et al., [11]	*	*			*	*	*	15%<br>18%	*	*	*	*	*	++
Hosseini-Amiri et al., [12]	*	*			*	*	*	0%<br>0%	*	*	+	*	*	++
Hunter et al., [13]	*	*			*	*	*	15%<br>8%	*	*	+	*	*	++
Krukowski et al., [14]	*	*			*	*	*	23%<br>38%	*	-	+	*	*	++
Lutes et al., [3]	*	*			*	*	*	9%<br>20%	*	*	++	*	*	++
McDoniel et al., [15]	*	*			*	*	*	24%<br>14%	*	-	+	*	*	+
Parastouei et al., [16]	*	*		*	*	*	*	0%<br>0%		-	+	*	*	+
Paravidino et al., [17]	*	*			*	*	*	0%<br>0%	*	-	+	*	*	+
Perez-Munoz et al., [18]	*	*			*	*	*	8%<br>13%	*	*	++	*	*	++
Smith et al.,[19]	*	*			*	*	*	0%<br>0%	*	-	*	*	*	++
Smith et al.,[20]	*	*	*		*	*	*	0%<br>0%	*	-	-	*	*	+
Staudter et al., [2]	*	*			*	*	*	30%<br>28%		-	-	*	*	+

Veverka et al., [21]	*	*			*	*	*	0%<br>0%		-	-	*	*		+
Voils et al.,[22]	*	*			*	*	*	20%<br>10%	*	*	++	*	*		++

Table S3. An overview of the interventions provided by each study

Study, Year	Behavioral and Lifestyle modifications	Diet or Nutritional modification	Self-monitoring	Specialistic counseling or non-clinician coaching provided	Internet-based	Pharmachological
Afari et al., [4]	X		X	X		
Boutelle et al., [5]	X		X	X		
Damschroder et al., [7]	X		X	X		
Dennis et al., [8]	X	X	X			
Erickson et al., [1]	X	X		X		
Evans-Hudnall et al., [9]	X			X		
Goldberg et al., [10]	X	X		X		
Hoerster et al., [11]	X		X		X	
Hosseini-Amiri et al., [12]	X		X			
Hunter et al., [13]	X	X	X		X	
Krukowski et al., [14]	X	X		X	X	
Lutes et al., [3]	X			X		
McDoniel et al., [15]	X	X		X		
Parastouei et al., [16]		X		X		
Paravidino et al., [17]	X		X			
Perez-Munoz et al., [18]	X	X	X			
Smith et al., [19]		X		X		
Smith et al., [20]	X	X		X		X
Staudter et al., [2]	X		X		X	
Veverka et al., [21]	X	X	X		X	
Voils et al., [22]	X	X	X			

Table S4. An overview of studies included in the meta-regression analyses

Study	BW longitudinal			BMI longitudinal		
	Age	*Body weight at baseline*	*Duration intervention*	*Age*	*BMI at baseline*	*Duration intervention*
Afari et al.,**[4]**						
Boutelle et al.,**[5]**						
Damschroder et al., **[7]**	X	X	X	X	X	X
Dennis et al.,**[8]**	X	X	X	X	X	X
Erickson et al., **[1]**						
Evans-Hudnall et al.,**[9]**	X	X	X			
Goldberg et al., **[10]**	X	X	X			
Hoerster et al., **[11]**	X	X	X			
Hosseini-Amiri et al.,**[12]**	X	X	X	X	X	X
Hunter et al.,**[13]**	X	X	X	X	X	X
Krukowski et al., **[14]**	X	X	X	X	X	X
Lutes et al.,**[3]**						
McDoniel et al., **[15]**	X	X	X	X	X	X
Parastouei et al., **[16]**				X	X	X
Paravidino et al., **[17]**	X	X	X	X	X	X
Perez-Munoz et al., **[18]**	X	X	X	X	X	X
Smith et al.,**[19]**	X	X	X	X	X	X
Smith et al.,**[20]**		X	X		X	X
Staudter et al., **[2]**						
Veverka et al., **[21]**		X	X		X	X
Voils et al.,**[22]**	X	X	X			

Table S5. Results for separate meta-analyses divided by intervention type

	**BW longitudinal** meta-analysis (pre-to-post)	**BW cross-sectional** meta-analysis (treat. vs contr.)	**BMI longitudinal** meta-analysis (pre-to-post)	**BMI cross-sectional** meta-analysis (treat. vs contr.)
**Behavioral & Lifestyle**	n= 14 studies<br>g= -0.010<br>95% CI: -0.18, -0.01<br>*p*=0.024	n= 14 studies<br>g= -0.08<br>95% CI: -0.20, 0.03<br>*p*=0.149	n= 10 studies<br>g= -0.28<br>95% CI: -0.45, -0.11<br>*p*=0.001	n= 10 studies<br>g= -0.18<br>95% CI: -0.28, -0.08<br>*p*<0.001
**Diet & Nutritional**	n= 10 studies<br>g= -0.09<br>95% CI: -0.21, 0.04<br>*p*=0.178	n= 10 studies<br>g= -0.09<br>95% CI: -0.20, 0.01<br>*p*=0.078	n= 9 studies<br>g= -0.30<br>95% CI: -0.50, -0.11<br>*p*=0.002	n= 9 studies<br>g= -0.15<br>95% CI: -0.26, -0.03<br>*p*=0.010
**Self-Monitoring**	n= 9 studies<br>g= -0.10<br>95% CI: -0.22, 0.02<br>*p*=0.103	n= 9 studies<br>g= -0.07<br>95% CI: -0.17, 0.04<br>*p*=0.214	n= 7 studies<br>g= -0.260<br>95% CI: -0.48, -0.04<br>*p*=0.021	n= 7 studies<br>g= -0.17<br>95% CI: -0.28, -0.06<br>*p*=0.003
**Counseling Provided**	n= 7 studies<br>g= -0.14<br>95% CI: -0.28, 0.00<br>*p*=0.058	n= 7 studies<br>g= -0.11<br>95% CI: -0.34, 0.11<br>*p*=0.321	n= 6 studies<br>g= -0.30<br>95% CI: -0.47, -0.14<br>*p*<0.001	n= 6 studies<br>g= -0.13<br>95% CI: -0.29, 0.03<br>*p*=0.113
**Internet-Based**	n= 4 studies<br>g= -0.16<br>95% CI: -0.28, -0.05<br>*p*=0.006	n= 4 studies<br>g= -0.14<br>95% CI: -0.26, -0.02<br>*p*=0.024	n= 3 studies<br>g= -0.22<br>95% CI: -0.37, -0.07<br>*p*=0.004	n= 3 studies<br>g= -0.25<br>95% CI: -0.40, -0.09<br>*p*=0.002

Abbreviations: n=number of studies; g= Hedge's g Effect Size; p= p-value; 95% CI: Confidence Interval

Figure S1. Forest Plot for the BW longitudinal meta-analysis in active-duty military personnel [8, 12-15,17-21]:

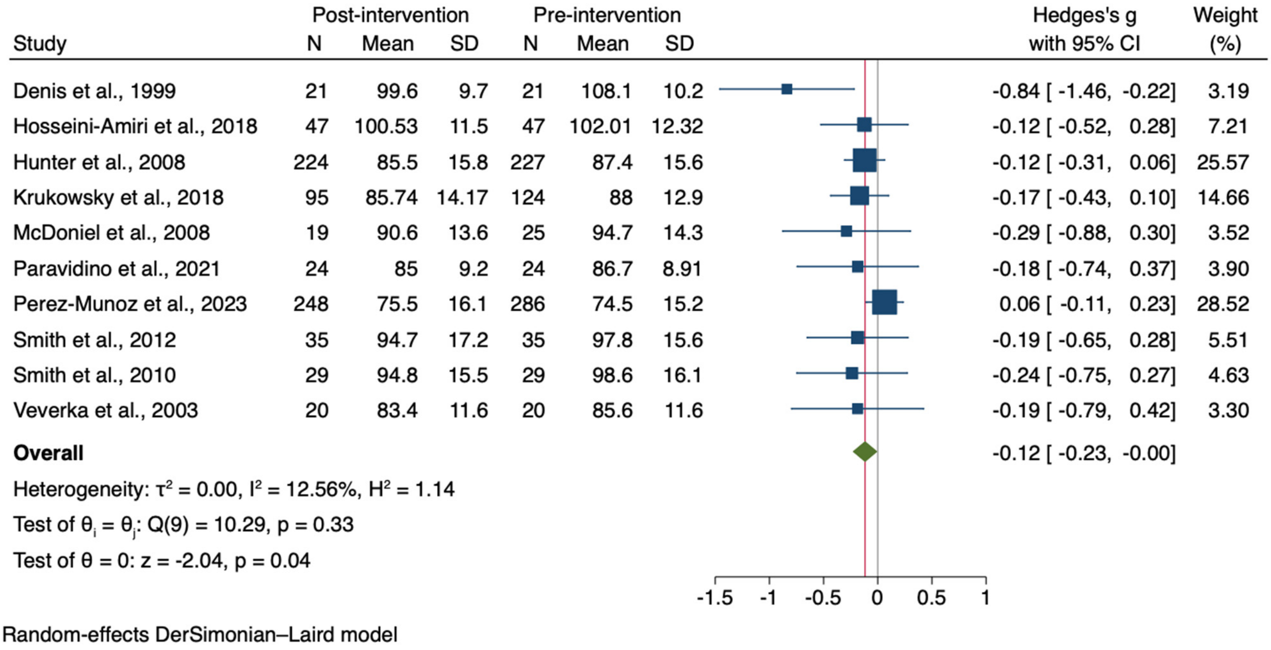

Figure S2. Forest Plot for the BMI longitudinal meta-analysis in active-duty military personnel [8,12-21]:

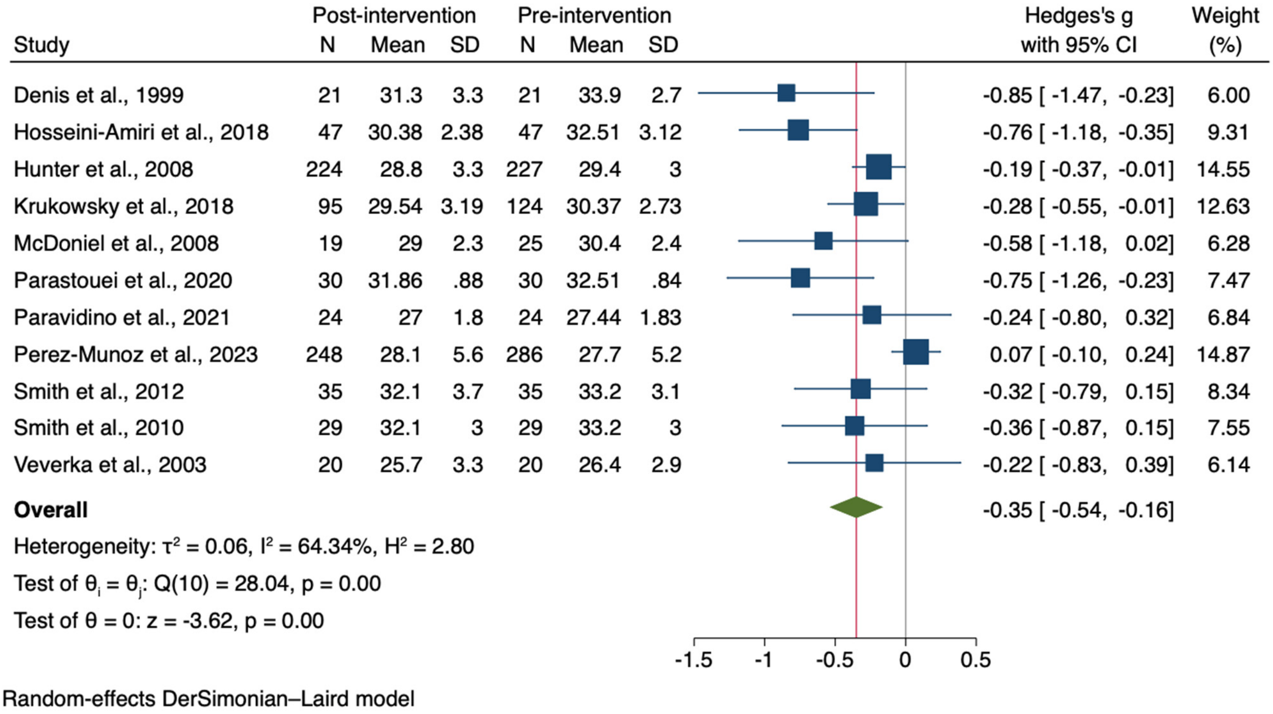

Figure S3. Forest Plot for the BW cross-sectional meta-analysis in active-duty military personnel [8, 12-15, 17-21]:

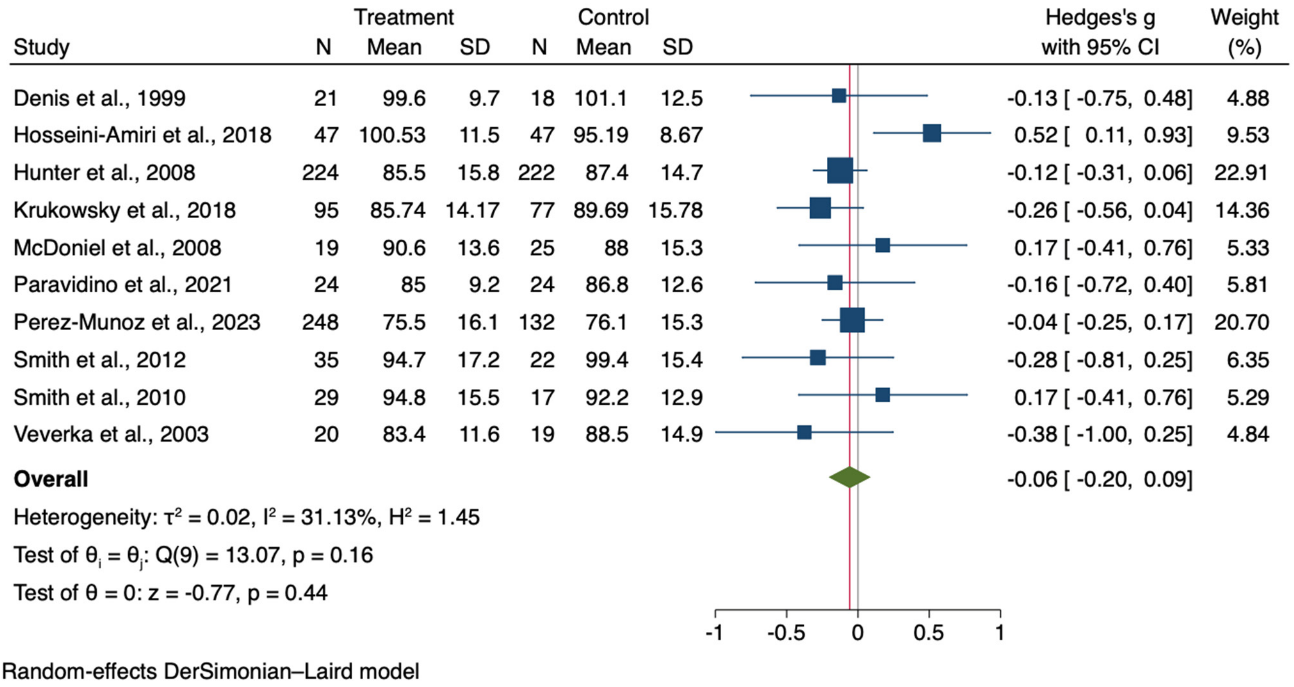

Figure S4. Forest Plot for the BMI cross-sectional meta-analysis in active-duty military personnel [8, 12-21]:

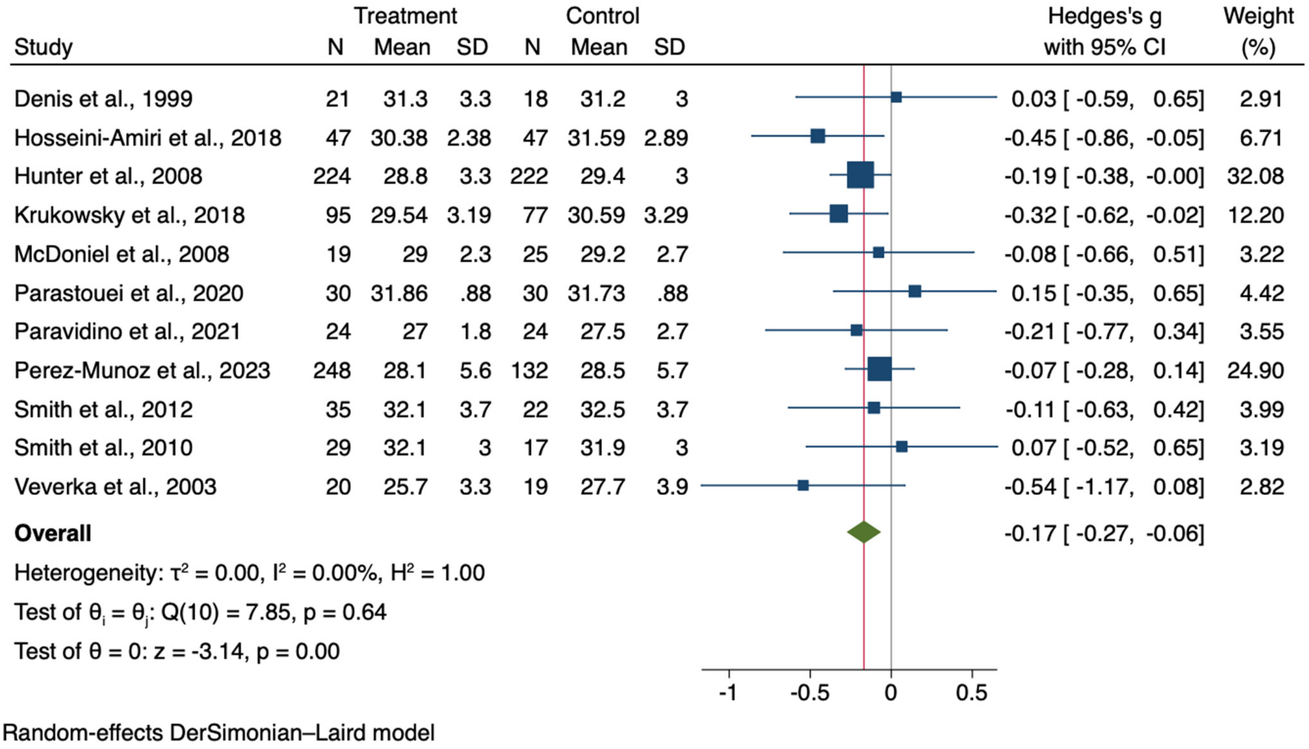

Figure S5. Forest Plot for the BW longitudinal meta-analysis in veterans [7, 9-11, 22]:

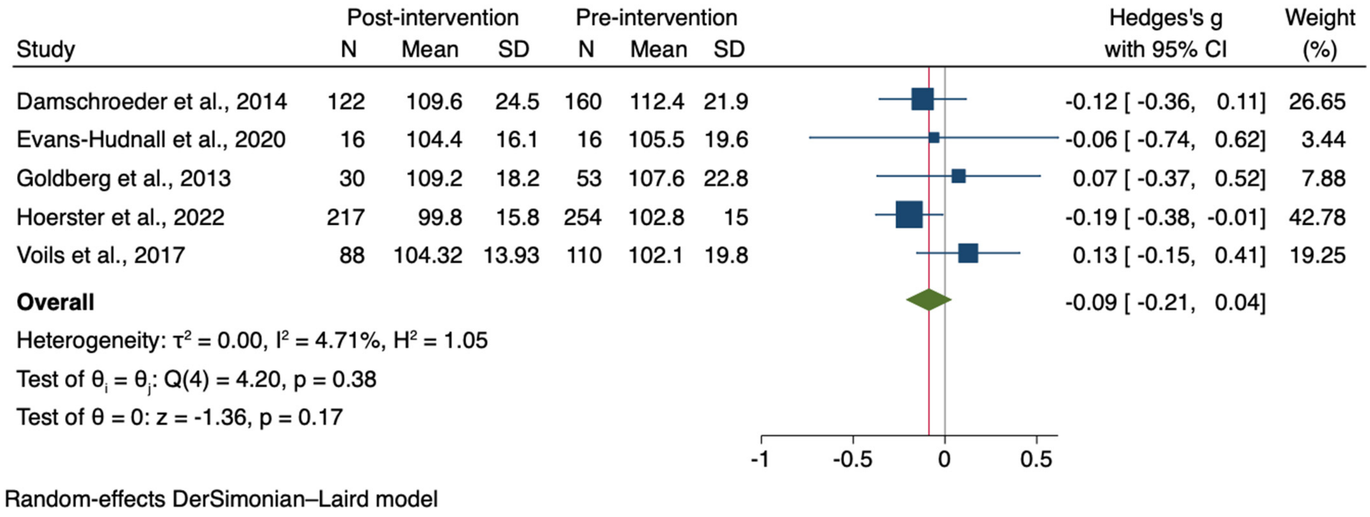

Figure S6. Forest Plot for the BW cross-sectional meta-analysis in veterans. [7, 9-11-22]:

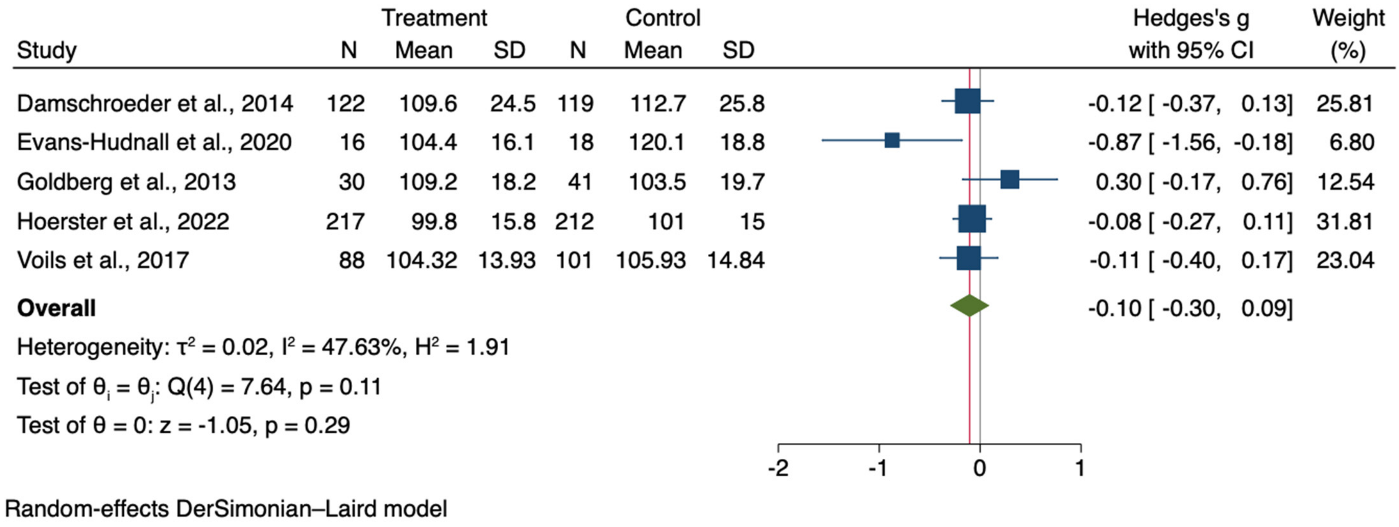

Figure S7. Funnel plot for the BW longitudinal meta-analysis.

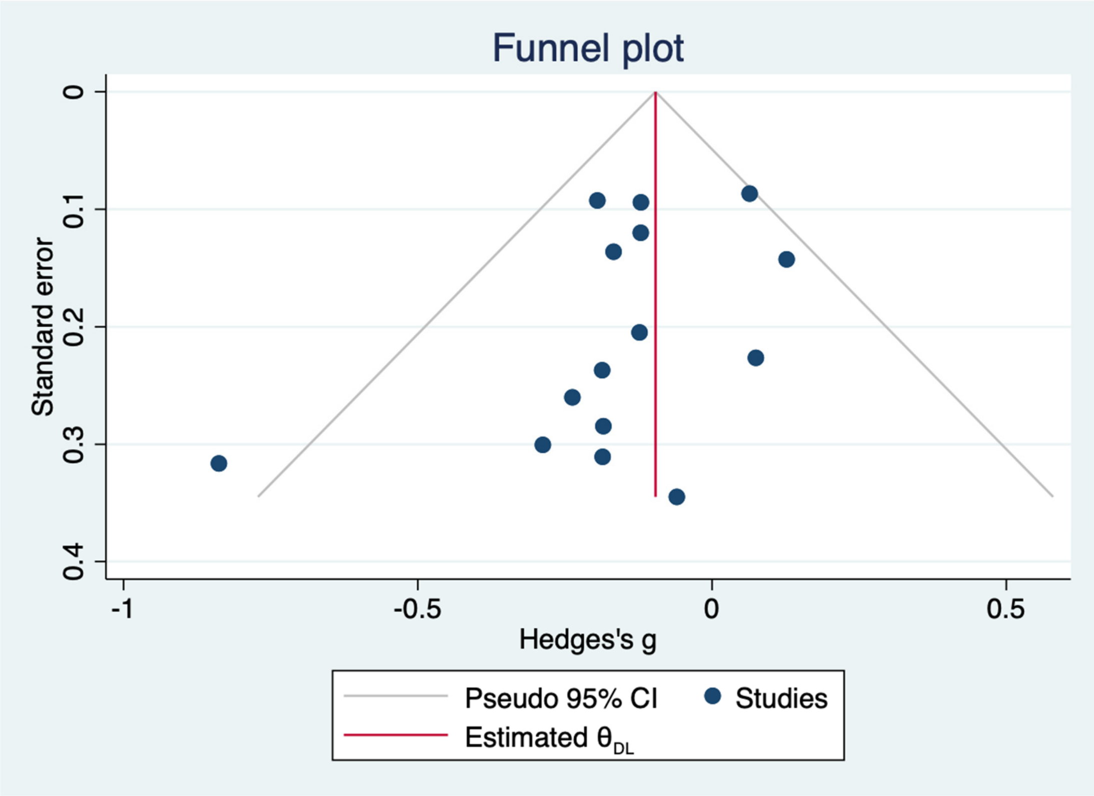

Figure S8. Funnel plot for the BMI longitudinal meta-analysis.

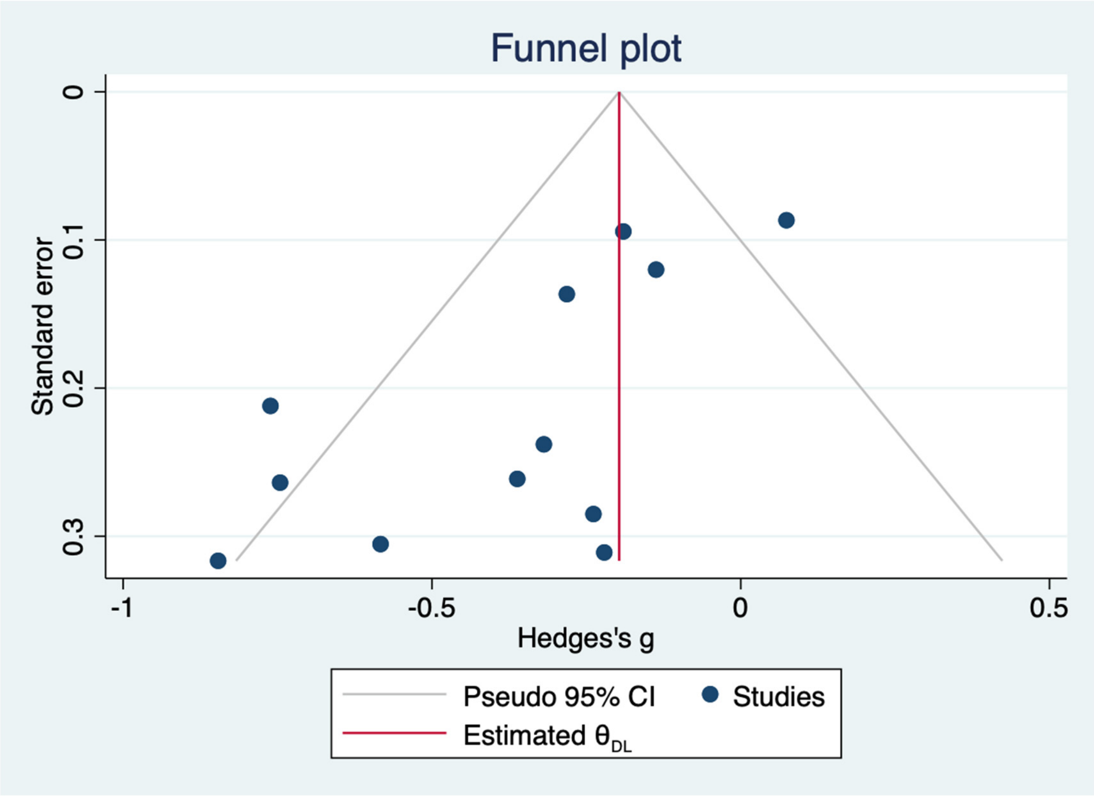

Figure S9. Funnel plot for the BW cross-sectional meta-analysis.

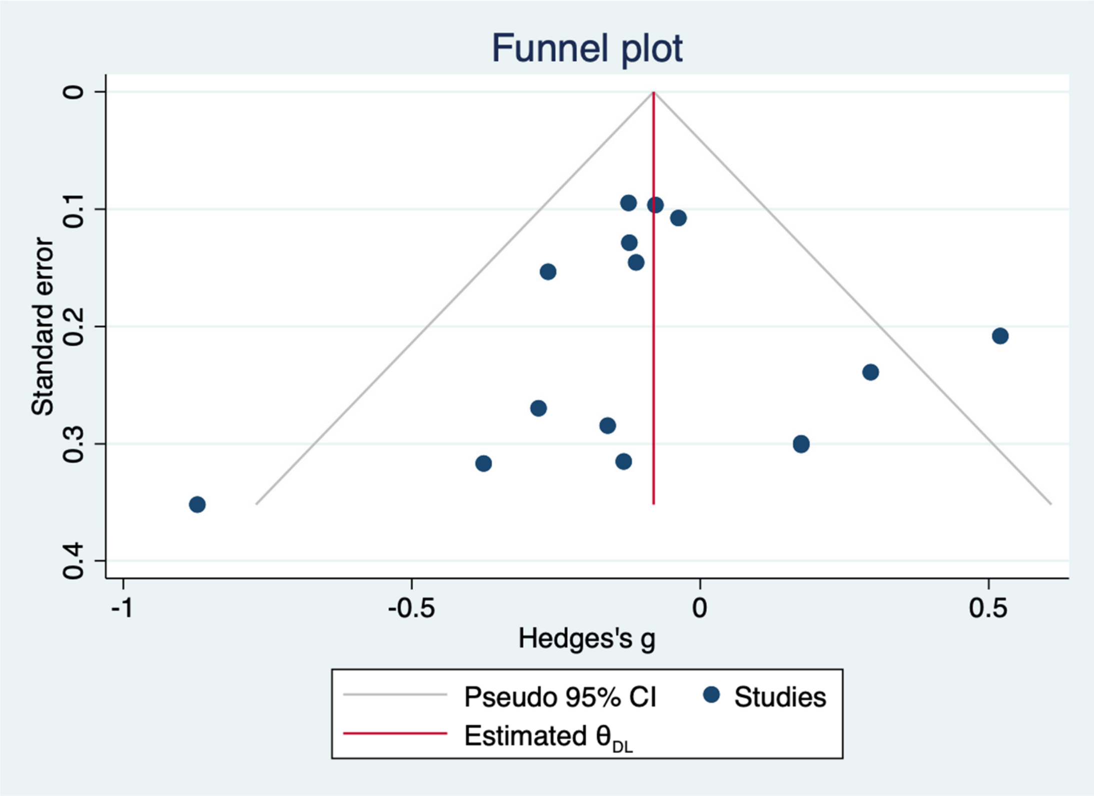

Figure S10. Funnel plot for the BMI cross-sectional meta-analysis.

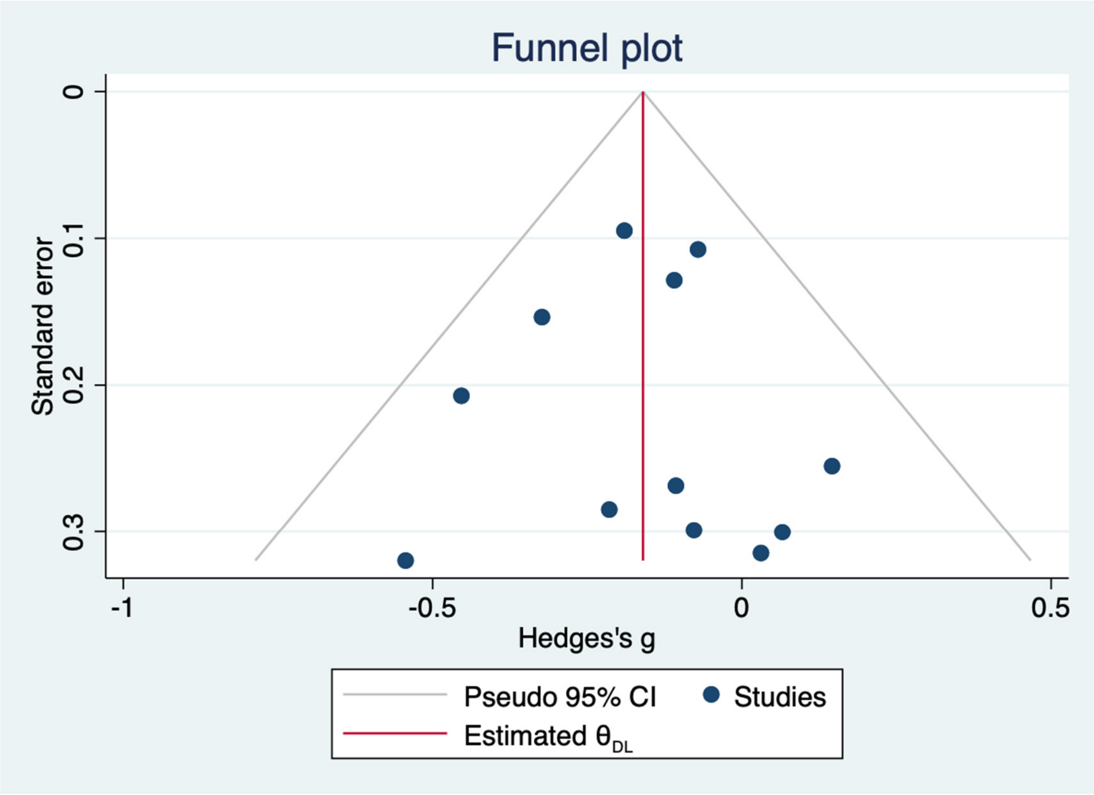

Figure S11. Funnel plot adjusted for publication bias for the BW longitudinal meta-analysis in active-duty military personnel, with imputed studies (n=2).

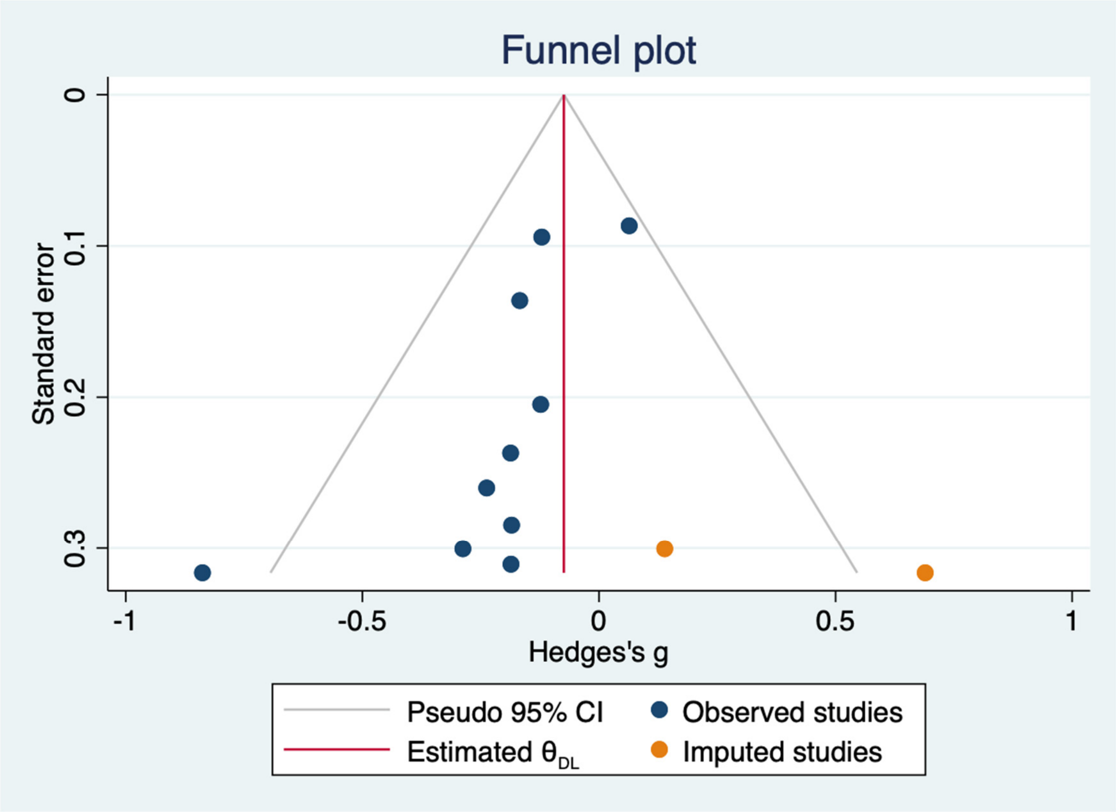

Figure S12. Funnel plot adjusted for publication bias for the BMI longitudinal meta-analysis in active-duty military personnel, with imputed studies (n=5).

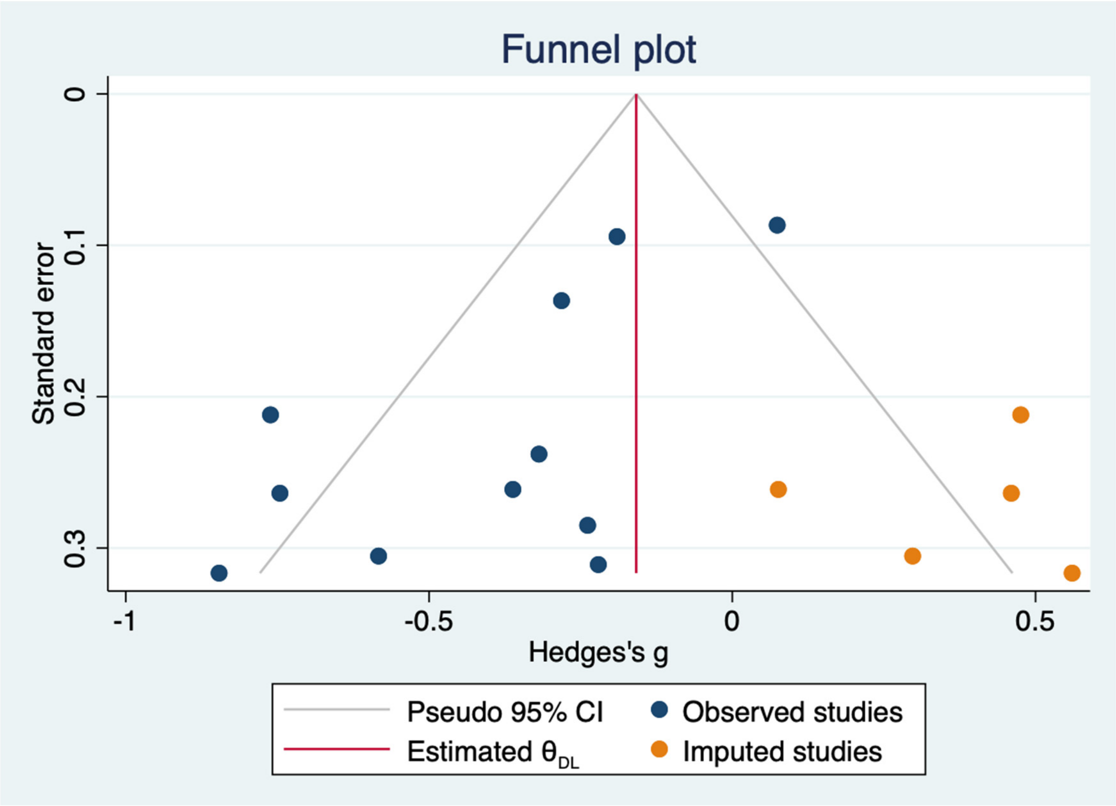

Figure S13. Funnel plot for the BW cross-sectional meta-analysis in active-duty military personnel.

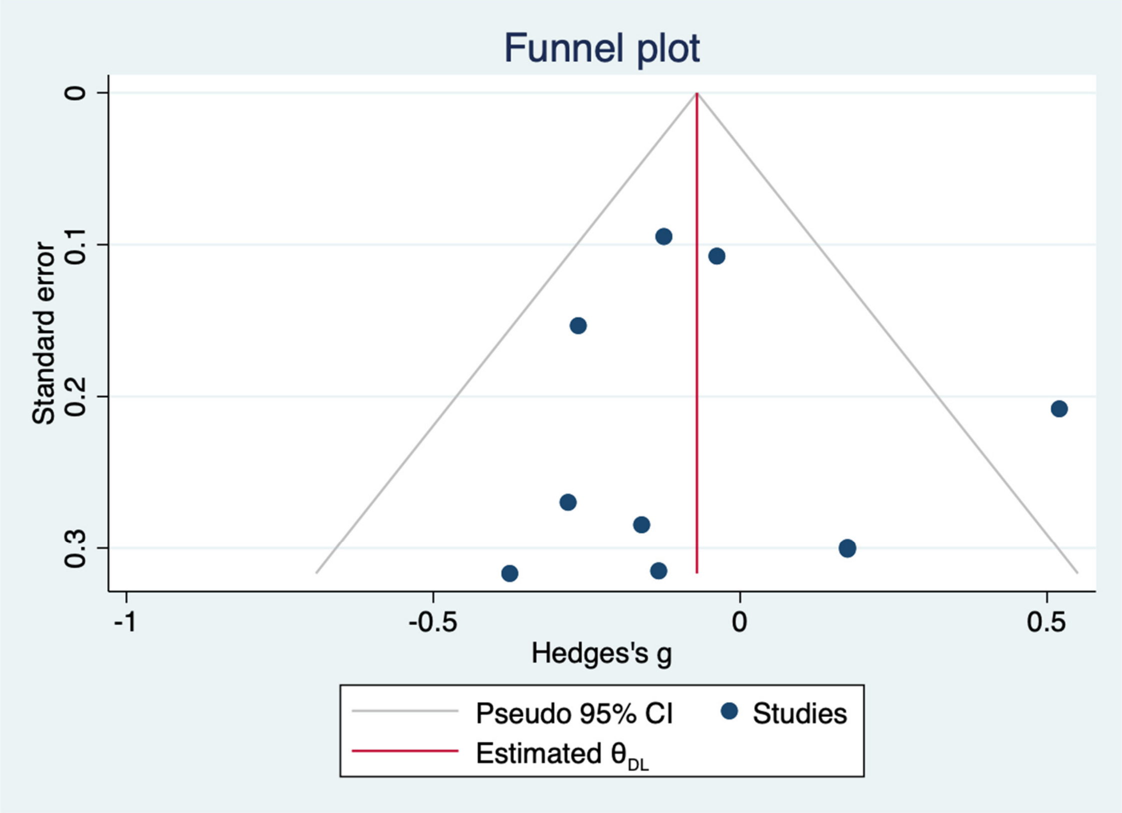

Figure S14. Funnel plot adjusted for publication bias for the BMI cross-sectional meta-analysis in active-duty military personnel, with imputed study (n=1).

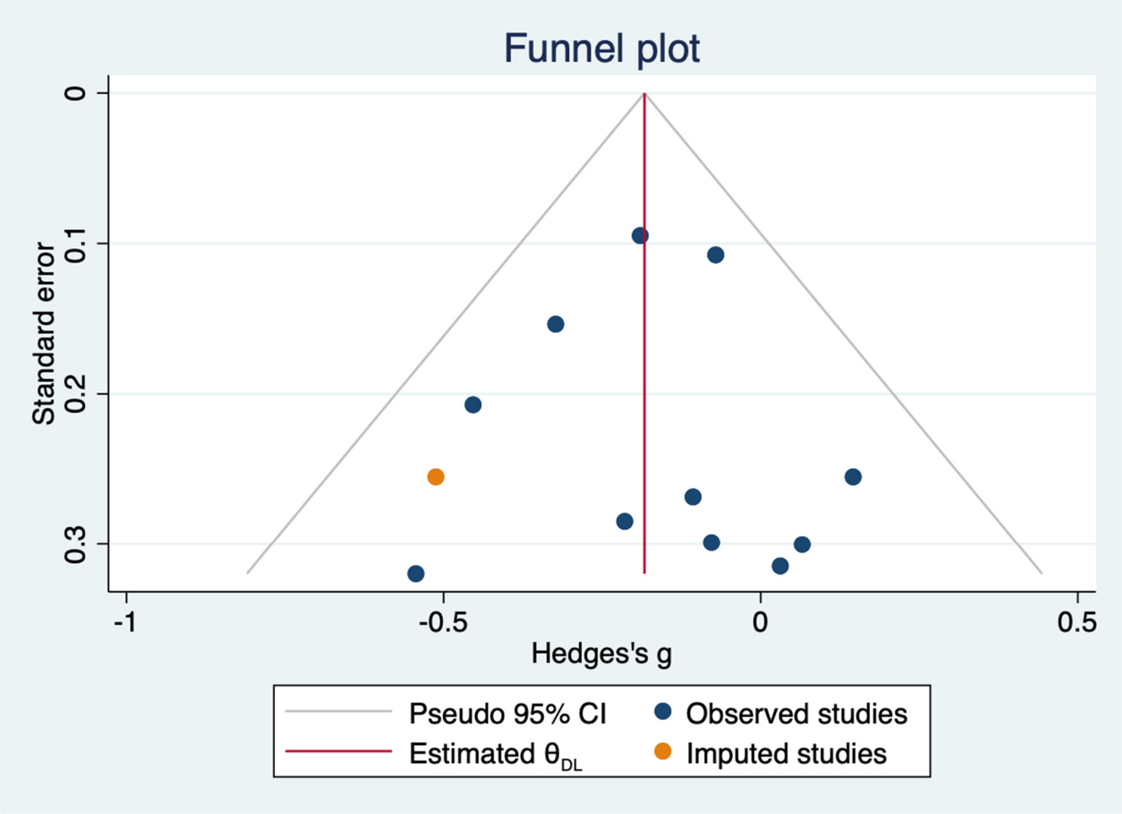

Figure S15. Funnel plot adjusted for publication bias for the BW longitudinal meta-analysis in veterans, with imputed study (n=3).

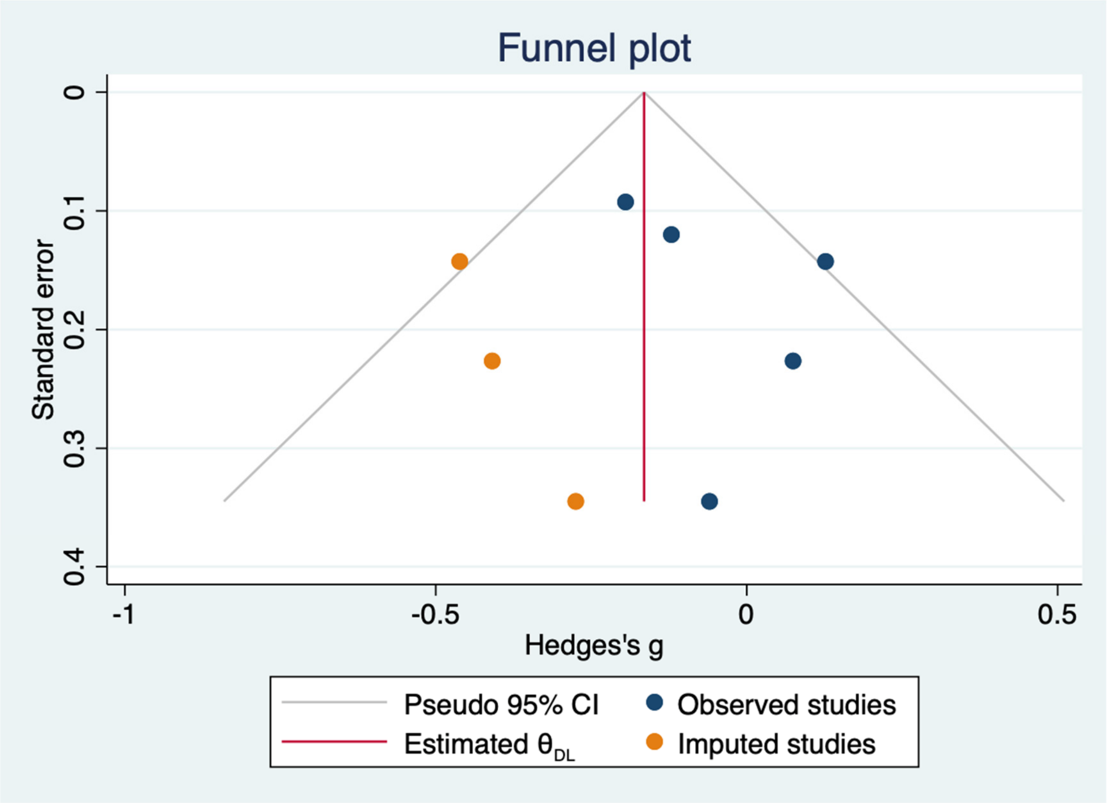

Figure S16. Funnel plot adjusted for publication bias for the BW cross-sectional meta-analysis in veterans.

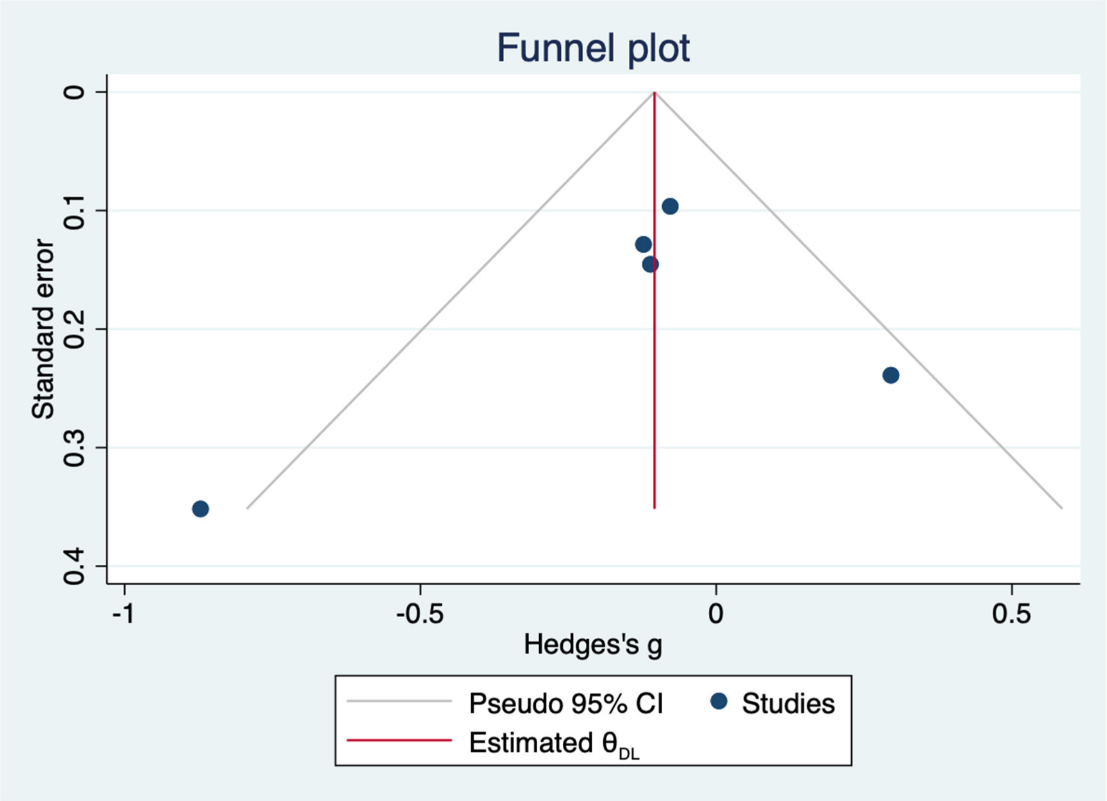

References

1. Erickson, Z.D.; Kwan, C.L.; Gelberg, H.A.; Arnold, I.Y.; Chamberlin, V.; Rosen, J.A.; Shah, C.; Nguyen, C.T.; Hellemann, G.; Aragaki, D.R.; et al. A randomized, controlled multisite study of behavioral interventions for veterans with mental illness and antipsychotic medication-associated obesity. J. Gen. Intern. Med. 2017, 32, 32–39.

2. Staudter, M.; Dramiga, S.; Webb, L.; Hernandez, D.; Cole, R. Effectiveness of pedometer use in motivating active duty and other military healthcare beneficiaries to walk more. US Army Med. Dep. J. 2011, Volume Jul-Sep: 108–119.

3. Lutes, L.D.; Damschroder, L.J.; Masheb, R.; Kim, H.M.; Gillon, L.; Holleman, R.G.; Goodrich, D.E.; Lowery, J.C.; Janney, C.; Kirsh, S.; et al. Behavioral treatment for veterans with obesity: 24-month weight outcomes from the ASPIRE-VA small changes randomized trial. J. Gen. Intern. Med. 2017, 32, 40–47.

4. Afari, N.; Cuneo, J.G.; Herbert, M.; Miller, I.; Webb-Murphy, J.; Delaney, E.; Peters, J.; Materna, K.; Miggantz, E.; Godino, J.; et al. Design for a cohort-randomized trial of an acceptance and commitment therapy-enhanced weight management and fitness program for Navy personnel. Contemp. Clin. Trials Commun. 2019, 15, 100408.

5. Boutelle, K.N.; Afari, N.; Obayashi, S.; Eichen, D.M.; Strong, D.R.; Peterson, C.B. Design of the CHARGE study: A randomized control trial evaluating a novel treatment for Veterans with binge eating disorder and overweight and obesity. Contemp. Clin. Trials 2023, 130, 107234.

6. Methodology checklist 2: randomised controlled trials. SIGN, Retrieved March 3, 2014, available from: https://www.sign.ac.uk/what-we-do/methodology/checklists

7. Damschroder, L.J.; Lutes, L.D.; Kirsh, S.; Kim, H.M.; Gillon, L.; Holleman, R.G.; Goodrich, D.E.; Lowery, J.C.; Richardson, C.R. Small-Changes Obesity Treatment among Veterans 12-Month Outcomes. Am. J. Prev. Med. 2014, 47, 541–553.

8. Dennis, K.E.; Pane, K.W.; Adams, B.K.; Qi, B.B. The impact of a shipboard weight control program. Obes. Res. 1999, 7, 60–67.

9. Evans-Hudnall, G.; O Odafe, M.; Johnson, A.; Armenti, N.; O'neil, J.; Lawson, E.; Trahan, L.H.; Rassu, F.S. Using an Adjunctive Treatment to Address Psychological Distress in a National Weight Management Program: Results of an Integrated Pilot Study. Mil. Med. 2020, 185, E1662–E1670.

10. Goldberg, R.W.; Reeves, G.; Tapscott, S.; Medoff, D.; Dickerson, F.; Goldberg, A.P.; Ryan, A.S.; Fang, L.J.; Dixon, L.B.; Muralidharan, A.; et al. "MOVE!": Outcomes of a weight loss program modified for veterans with serious mental illness. Psychiatr. Serv. 2013, 64, 737–744.

11. Hoerster, K.D.; Hunter-Merrill, R.; Nguyen, T.; Rise, P.; Barón, A.E.; McDowell, J.; Donovan, L.M.; Gleason, E.; Lane, A.; Plumley, R.; et al. Effect of a Remotely Delivered Self-directed Behavioral Intervention on Body Weight and Physical Health Status among Adults with Obesity: The D-ELITE Randomized Clinical Trial. JAMA 2022, 328, 2230–2241.

12. Hosseini-Amiri, M.; Aliyari, S.; Zareiyan, A.; Dabbagh-Moghadam, A. The Effects of Extended Parallel Process Model on Obese Soldiers' Knowledge, Attitudes, and Practices about Obesity Management: A Randomized Controlled Clinical Trial. Iran. J. Nurs. Midwifery Res. 2018, 23, 458–464.

13. Hunter, C.M.; Peterson, A.L.; Alvarez, L.M.; Poston, W.C.; Brundige, A.R.; Haddock, C.K.; Van Brunt, D.L.; Foreyt, J.P. Weight management using the internet: A randomized controlled trial. Am. J. Prev. Med. 2008, 34, 119–126.

14. Krukowski, R.A.; Hare, M.E.; Talcott, G.W.; Johnson, K.C.; Richey, P.A.; Kocak, M.; Balderas, J.; Colvin, L.; Keller, P.L.; Waters, T.M.; et al. Dissemination of the Look AHEAD Intensive Lifestyle Intervention in the United States Military: A Randomized Controlled Trial. Obesity 2018, 26, 1558–1565.

15. McDoniel, S.O.; Nelson, H.A.; Thomson, C.A. Employing RMR technology in a 90-day weight control program. Obes. Facts. 2008, 1, 298–304.

16. Parastouei, K.; Saeidipoor, S.; Sepandi, M.; Abbaszadeh, S.; Taghdir, M. Effects of synbiotic supplementation on the components of metabolic syndrome in military personnel: A double-blind randomised controlled trial. BMJ Mil. Health 2022, 168, 362–367.

17. Paravidino, V.B.; Mediano, M.F.F.; Silva, I.C.M.; Wendt, A.; Del Vecchio, F.B.; Neves, F.A.; Terra, B.d.S.; Gomes, E.A.C.; Moura, A.S.; Sichieri, R. Effect of physical exercise on spontaneous physical activity energy expenditure and energy intake in overweight adults (the EFECT study): A study protocol for a randomized controlled trial. Trials 2018, 19, 167.

18. Pérez-Muñoz, A.; Hare, M.E.; Andres, A.; Klesges, R.C.; Wayne Talcott, G.; Little, M.A.; Waters, T.M.; Harvey, J.R.; Bursac, Z.; Krukowski, R.A. A Postpartum Weight Loss-focused Stepped-care Intervention in a Military Population: A Randomized Controlled Trial. Ann. Behav. Med. 2023, 2023, kaad014.

19. Smith, T.J.; Sigrist, L.D.; Bathalon, G.P.; McGraw, S.; Karl, J.P.; Young, A.J. Efficacy of a meal-replacement program for promoting blood lipid changes and weight and body fat loss in US Army soldiers. J. Am. Diet. Assoc. 2010, 110, 268–273.

20. Smith, T.J.; Crombie, A.; Sanders, L.F.; Sigrist, L.D.; Bathalon, G.P.; McGraw, S.; Young, A.J. Efficacy of orlistat 60 mg on weight loss and body fat mass in US Army soldiers. J. Acad. Nutr. Diet. 2012, 112, 533–540.

21. Veverka, D.V.; Anderson, J.; Auld, G.W.; Coulter, G.R.; Kennedy, C.; Chapman, P.L. Use of the stages of change model in improving nutrition and exercise habits in enlisted Air Force men. Mil. Med. 2003, 168, 373–379.

22. Voils, C.I.; Olsen, M.K.; Gierisch, J.M.; McVay, M.A.; Grubber, J.M.; Gaillard, L.; Bolton, J.; Maciejewski, M.L.; Strawbridge, E.; Yancy, W.S., Jr. Maintenance of weight loss after initiation of nutrition training: A randomized trial. Ann. Intern. Med. 2017, 166, 463–471.
